# Imputation of missing information in worldwide patent data

**DOI:** 10.1016/j.dib.2020.106615

**Published:** 2020-12-05

**Authors:** Gaétan de Rassenfosse, Florian Seliger

**Affiliations:** a Chair of Innovation and Intellectual Property Policy, College Management of Technology, Ecole polytechnique fédérale de Lausanne Switzerland; b KOF Swiss Economic Institute, Department of Management, Technology, and Economics, ETH Zurich. NZZ Media Group, Zurich, Switzerland

**Keywords:** Missing data, Patents, PATSTAT, Imputation, PostgreSQL

## Abstract

We present a general method for imputing missing information in the Worldwide Patent Statistical Database (PATSTAT) and make the resulting datasets publicly available. The PATSTAT database is the de facto standard for academic research using patent data. Complete information on patents is essential to obtain an accurate picture of technological activities across countries and over time. However, the coverage of the database is far from complete. Our data imputation method exploits detailed institutional knowledge about the international patent system, and we codify it in a SQL algorithm. We provide two datasets related to the imputation of missing country codes and missing technology classification. We also release the algorithm that can be easily adapted to impute other pieces of information that are missing in PATSTAT.

## Specifications Table

**Table:** 

Subject	Social Sciences (General)
Specific subject area	Innovation policies, regional studies, strategic management
Type of data	Table Dataset PostgreSQL code
How data were acquired	The data were extracted from the Worldwide Patent Statistical Database (PATSTAT)
Data format	Raw
Parameters for data collection	PATSTAT needs to be set up as a PostgreSQL database
Description of data collection	The datasets result from querying different PATSTAT tables, extracting the desired information and inserting it into an output table
Data source location	European Patent Office, Vienna
Data accessibility	Repository name: Harvard Dataverse Data identification number: https://doi.org/10.7910/DVN/U5BUCT, https://doi.org/10.7910/DVN/XNTL0W, https://doi.org/10.7910/DVN/NTSV0L Direct URL to data: https://dataverse.harvard.edu/dataverse/imputation_worldwide_patent_data

## Value of the Data

• The Worldwide Patent Statistical Database PATSTAT, provided by the European Patent Office (EPO), has become the de facto standard for researchers working with patent data. A critical issue with the database, however, is that its coverage is far from being complete.
• Complete patent data are crucial to delivering an accurate picture of innovation activities around the globe. Researchers and policymakers use patent data for many purposes, but often refer to a selected set of patent offices or to incomplete data. By using our data / our suggested imputation method, a more accurate picture of innovation activities can be obtained.
• Patents in the same family offer an abundant reservoir of information to fill in potential missing data. We provide a systematic approach to replenish missing pieces of information by browsing different pools of subsequent filings. The code can be easily adapted to other cases of missing information in patent data.
• In general, our data show the usefulness of imputation and the need to reflect carefully on all selection decisions when working with patent data.

## 1 Data Description

We draw on de Rasssenfosse et al. (2013, 2019) [1,2] who argue that the first filing of a patent family for a given invention is the relevant entity to look at. Indeed, first filings are the first occurrences of the invention, and, loosely speaking, second filings correspond to 'replicates' of the first filings that extend patent protection in other jurisdictions. If any information is missing for the first filing, it is possible to infer it from the subsequent filings in the same family. Our algorithm detects the data gaps in the first filings and browses the relevant subsequent filings in order to fill in the missing information.

We provide three different datasets in our Dataverse (https://dataverse.harvard.edu/dataverse/imputation_worldwide_patent_data) where we have applied the algorithm in order to provide complete data on country codes of inventors and applicants and technology classification:

1. Imputation of missing technology classification in worldwide patent data.
2. Imputation of missing applicant country codes in worldwide patent data.
3. Imputation of missing inventor country codes in worldwide patent data.

All files contain application identifiers for first filings (corresponding to APPLN_ID in PATSTAT), the first filing date and year, and a column with the desired information (1. technology classification, 2. applicant country codes, or 3. inventor country codes, for details see below). The TYPE column indicates the type of the first filing (see next section). Datasets 1. and 2. also contain a PERSON_ID. This is also a PATSTAT ID that can be used to identify inventors and applicants and to join more detailed address information using the respective PATSTAT tables.

The datasets are zipped. The unzipped files are very large (between 3 and 11GB) and cannot be opened with conventional text editors or spreadsheet software. For inspecting the files, EmEditor – a text editor for Windows that supports large amounts of data – can be used. For Mac users there are suitable alternatives that can be found in the World Wide Web. In any case, we suggest using a SQL database.

Missing information have been imputed from equivalents and other second filings (see next section). The SOURCE column indicates the respective source of information.

The information is directly retrieved from the relevant PATSTAT tables. Details, definitions and links to references can be found in the PATSTAT data catalog [3]. In particular, for the data mentioned above, we have retrieved the following fields from PATSTAT:

1. Everything related to technology classification, in particular the International Patent Classification (IPC), the Cooperative Patent Classification (CPC), and technology fields that are more aggregated and have been derived from the IPC.
  • IPC_CLASS_LEVEL: Denotes whether an authority classified either in the full IPC, in main groups or in subclasses only.
  • IPC_CLASS_SYMBOL and CPC_CLASS_SYMBOL: Classification symbol according to the International Patent Classification (IPC) / Cooperative Patent Classification (CPC).
  • IPC_GENER_AUTH and CPC_GENER_AUTH: Patent office that generated the IPC classification of the application concerned / patent office that classified the application with a CPC symbol.
  • IPC_MAINGROUP_SYMBOL: The subclass (i.e. first 4 characters) or main group (i.e. first 8 characters) of an IPC symbol.
  • IPC_POSITION and CPC_POSITION: Indicates the position of the class symbol in the sequence of classes that form the classification (only relevant when patent authorities apply the concept of the “first” class, i.e. the first class symbol in a list of class symbols is the main class).
  • IPC_VALUE and CPC_VALUE: Indication of the value of the classification, i.e. if the class symbol is relating to the invention or to aspects not related to the invention.
  • IPC_VERSION and CPC_VERSION: Version of the IPC / CPC.
  • CPC_SCHEME: Indicates whether the CPC symbol has been allocated by the European Patent Office (EPO), the United States Patent and Trademark Office (USPTO) or a National Office.
  • TECHN_FIELD_NR: Uniquely identifies a technology field.
  • TECHN_FIELD: Name of a technology field.
  • WEIGHT: Weight of the association between the application and the IPC. The higher the number, the stronger the relationship between an application and a technical field.
  • TECHN_SECTOR: The technology fields are grouped in five broader technology sectors.
2. and **3** CTRY_CODE (corresponds to PERSON_CTRY_CODE in PATSTAT tables): Country part of the correspondence address of the person or business (inventor or applicant).

We codify the algorithm in SQL for PostgreSQL 9.6.6. The algorithm runs with any PATSTAT version newer than Autumn 2016, with only minor adaptations. However, careful inspection of the PATSTAT data catalog [3] of the respective PATSTAT version is warranted to adjust to changing data schema (minor changes such as relabeling of columns and tables can happen over time).

The SQL code can be found in our GitHub repositories:

1. https://github.com/seligerf/Imputation-of-missing-IPC-codes-and-technology-information-for-worldwide-patent-data
2. https://github.com/seligerf/Imputation-of-missing-location-information-for-worldwide-patent-data

There, you can also find code in order to build a “bridge” table in order to assign any patent filing to its respective first filing as defined in our work.

## 2 Experimental Design, Materials and Methods

PATSTAT's coverage suffers from two significant limitations:

1. *Inconsistency over time for some offices.* This problem concerns mainly small national patent offices from developing countries. A representative case is that of the Indonesian patent office, for which data are available from 1982 to 2001 (with one gap year), missing for 2002, 2003, and 2005–2011, and available afterward.
2. *Inconsistency within fields for some offices.* Even for patent offices for which the time coverage is satisfactory, important bibliographical information is missing for a significant proportion of patent filings. Fields for which information is often missing (especially for earlier years) comprise abstracts, technological classifications, citations, as well as applicant and inventor addresses and countries.

Table 1 provides an overview of data available for selected patent offices, years, and fields. The share of available inventor country codes for patent applications filed at the French patent office is lower than two percent before 2003, but almost complete starting in 2009. The situation is the reverse at the Chinese patent office. Concerning address data for inventors and applicants, only data from the EPO and USPTO are available on a large scale.

**Table 1:** Share of data available in different fields in PATSTAT for selected years at the largest patent offices.

Patent office	Year	No. Filings	Abstract	Applicant address	Applicant country	Citation	Inventor address	Inventor country	IPC	Legal Event
			*Percentage of information available*							
CA	1990	28402	100	59	87	0	0	100	100	100
CN	1990	9518	52	0	100	0	0	100	98	100
DE	1990	102954	20	0	93	20	0	91	100	71
EP	1990	63898	99	100	100	93	100	100	100	100
FR	1990	14582	100	0	77	92	0	1	100	82
GB	1990	28086	32	33	37	35	0	0	61	44
JP	1990	347106	95	0	0	17	0	0	100	25
KR	1990	14890	33	0	100	0	0	100	99	91
US	1990	99540	80	95	100	100	99	100	100	100
WO	1990	19078	100	100	100	100	100	100	100	100
CA	2000	42750	98	0	100	0	0	100	100	100
CN	2000	57560	58	0	98	0	0	97	100	100
DE	2000	108233	46	0	100	29	0	98	100	98
EP	2000	110729	54	100	100	99	100	100	100	100
FR	2000	15196	100	0	100	94	0	1	100	89
GB	2000	31725	41	0	43	40	0	42	61	96
JP	2000	372797	92	0	0	12	0	0	100	69
KR	2000	82130	87	0	71	6	0	74	91	100
US	2000	199450	100	92	97	98	96	100	100	100
WO	2000	91203	100	0	100	99	0	70	100	100
CA	2010	36176	97	0	100	0	0	100	100	100
CN	2010	361268	98	0	0	82	0	0	100	100
DE	2010	50734	94	0	100	82	0	96	100	99
EP	2010	133630	50	100	100	99	99	99	100	100
FR	2010	14270	100	0	100	94	0	100	100	93
GB	2010	21987	46	0	46	45	0	45	47	86
JP	2010	265346	88	0	0	71	0	0	100	83
KR	2010	158707	79	0	99	38	0	98	100	100
US	2010	323703	100	100	100	98	100	100	100	100
WO	2010	160839	100	0	100	100	0	96	100	100

``WO'' refers to the World Intellectual Property Office.

Calculations by EPO, source: https://public.tableau.com/profile/patstat.support#!/vizhome/CoverageofPATSTAT2019AutumnEdition/CoveragePATSTATGlobal, accessed on 2020/05/24

A useful feature of patent data in our context is that many patent applications *for the same invention* (or a close enough version of it) are filed in different jurisdictions, thus forming an international patent family. Therefore, the chances are high that information gaps on a focal patent can be retrieved from other members of the patent family. However, one needs detailed institutional knowledge about the patent system to understand how to fill these gaps accurately. The imputation algorithm that we propose implements a solution that exploits this knowledge. We publicly release it so that other scholars can replicate our approach, and possibly further refine it—or tailor it to specific use cases.

**Fig. 1:**
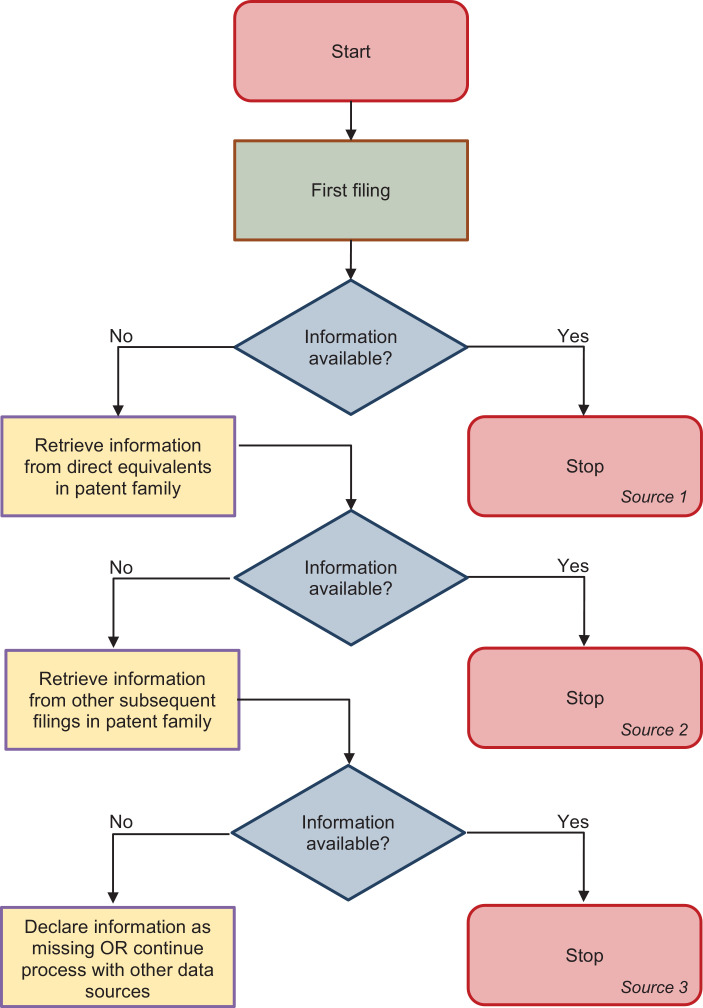
Flowchart of the algorithm.

Fig. 1 provides the algorithm's flowchart. The first step involves the creation of a table with all first filings of interest (regardless of whether the information is missing). First filings are the patent applications with the earliest application filing date within a patent family at any patent office. By default, we include first filings from patent offices from all OECD countries, including all EU28 countries (+ Switzerland and Norway), BRICS countries, the EPO, and the ‘International Bureau’ of the World Intellectual Property Office (WIPO). Patent applications filed at those offices account for almost all patent activity around the world. The ‘pool of first filings’ constitutes *source 1*.

The identification of first filings requires detailed knowledge of PATSTAT and the patent system. We gather first filings from PATSTAT in the broadest sense, i.e., all filings that have been applied for the first time for a given invention. First, we use all priority filings as defined in the strict sense, namely the 'Paris Convention’ priorities. The 1883 Paris Convention for the Protection of Industrial Property allows the applicant of a first application filed in one of the contracting states to seek protection in any of the other contracting states within 12 months. We also added Patent Cooperation Treaty (PCT) filings to our pool of first filings. The PCT makes it possible to seek patent protection in a large number of countries simultaneously. Finally, we can identify two other kinds of first filings: 'Parent applications' of so-called 'Application continuations'; and filings based on 'Technical relations' that define some kind of family-relationship. The PATSTAT data catalog offers technical definitions [3], more details can be also found in de Rassenfosse et al. (2019) [2]. We have included a TYPE column in our data so that it is possible to select specific types of first filings, e.g. only priority filings filed according to the Paris Convention.

Next, we create several tables that contain all necessary information to be used in the imputation when the information is not available from *source 1*. The imputation exploits the pool of all subsequent filings that relate to the first filings. Subsequent filings are patent applications filed in other jurisdictions than the first filing (except for continuals and technical relationships that do not constitute international patent families). In the case of PCT applications, we refer to information from the National or Regional Phase, where the applicant seeks protection at national or regional offices. If the information is not directly available (*source 1*), the algorithm will first look into direct equivalents of the first filing (*source 2*). These are subsequent filings that refer to exactly one first filing in a given office.^1^ The number of first filings they refer to can be retrieved from the PATSTAT tables mentioned above. If several equivalents exist, we select the equivalent with the earliest filing date.

If the information is not available from equivalents, the algorithm will look into other subsequent filings (*source 3*) and again select the filing with the earliest filing date. If the information cannot be retrieved from *source 3*, it is declared missing, i.e., the respective patent filing cannot be used in the statistical analysis.

The algorithm browses all sources step by step and inserts the information into a table that contains all application identifiers of first filings (APPLN_ID). It also stores the source of information (*source 1* to *3*) in a dedicated column (SOURCE). The resulting table can be used immediately for statistical analysis and can be easily combined with other PATSTAT data.

We provide two examples to illustrate the main imputation mechanisms and discuss potential problems in the results: imputation of missing information for inventor country codes and technology codes (IPC classification).

### 2.1 Imputation of inventor country codes

This example replicates results presented in de Rassenfosse et al1. (2013) [1]. Table 2 shows the proportion of information for first filings that is available before and after imputation. As we can see, the coverage improves significantly, especially for patent offices where the share of available information is meager to start with. For example, for France, the share of available information before imputation amounts to 1.1 percent in 1990 but reaches 49.8 percent after imputation. However, even after imputation, the numbers for some countries are too low to be exploited (e.g., China 2.8% in 2010, Great Britain 27.5% in 1990). Of course, imputation from the patent family is only possible if a family exists which is not always the case.

**Table 2:** Share of available information for inventor countries before and after imputation (sources 1 to 3).

Patent office	Year	No. first filings	Inventor country before imputation (%)	Inventor country after imputation (%)
CA	1990	4832	99.6	99.6
CN	1990	28289	99.7	99.7
DE	1990	31422	78.4	88.2
EP	1990	8605	99.4	99.4
FR	1990	11046	1.1	49.8
GB	1990	4451	0.9	27.5
JP	1990	317810	0.0	8.9
KR	1990	15069	100.0	100.0
US	1990	63210	99.9	99.9
WO	1990	3127	99.9	99.9
CA	2000	5014	98.9	99.6
CN	2000	76905	99.9	99.9
DE	2000	44793	99.9	99.9
EP	2000	11207	99.5	99.7
FR	2000	12999	0.6	60.0
GB	2000	6670	99.6	99.8
JP	2000	352646	0.0	13.0
KR	2000	84934	99.1	99.1
US	2000	130298	100.0	100.0
WO	2000	12198	85.2	90.8
CA	2010	2464	99.6	99.7
CN	2010	585934	0.0	2.8
DE	2010	38316	100.0	100.0
EP	2010	13705	99.6	99.8
FR	2010	13560	100.0	100.0
GB	2010	5456	99.3	99.7
JP	2010	241803	0.0	19.6
KR	2010	115917	90.4	94.7
US	2010	159334	100.0	100.0
WO	2010	23611	97.1	98.5

It is possible to further improve the recovery of data by continuing the imputation process using information on applicant country codes or patent office origin. Indeed, the country of the applicant is usually a good proxy for the country of the inventor. The same data recovery process can thus be performed, leading to *sources 4* to *6*. If information is still missing after browsing all these sources, the country code can be set equal to that of the patent office (source 7). In the data we provide, we browse all possible *sources 1* to *7*, i.e. for no filing the information is declared as missing and we can provide a country code for all filings in the database.

### 2.2 Imputation of IPC codes

The coverage of IPC codes is higher than for inventor country codes in the raw data, although there are still some gaps in recent years. However, after browsing *sources 2* and *3*, we obtain a 100 percent coverage for all patent offices’ first filings. Therefore, our algorithm allows researchers to draw on the complete pool of first filings from all important patent offices around the world when analyzing technological fields. Table 3 shows the proportion of information for first filings that is available before and after imputation.

**Table 3:** Share of available information on IPC before and after imputation.

Patent office	Year	No. first filings	IPC before imputation (%)	IPC after imputation (%)
CA	1990	4832	99.8	100.0
CN	1990	28289	99.9	100.0
DE	1990	31422	91.7	100.0
EP	1990	8605	93.9	100.0
FR	1990	11046	99.5	100.0
GB	1990	4451	86.5	100.0
JP	1990	317810	96.6	100.0
KR	1990	15069	95.7	100.0
US	1990	63210	72.5	100.0
WO	1990	3127	98.6	100.0
CA	2000	5014	98.9	100.0
CN	2000	76905	98.9	100.0
DE	2000	44793	90.6	100.0
EP	2000	11207	77.8	100.0
FR	2000	12999	98.4	100.0
GB	2000	6670	84.7	100.0
JP	2000	352646	92.5	100.0
KR	2000	84934	97.8	100.0
US	2000	130298	85.7	100.0
WO	2000	12198	98.2	100.0
CA	2010	2464	99.5	100.0
CN	2010	585934	98.8	100.0
DE	2010	38316	90.2	100.0
EP	2010	13705	68.6	100.0
FR	2010	13560	98.2	100.0
GB	2010	5456	55.7	100.0
JP	2010	241803	87.9	100.0
KR	2010	115917	96.2	100.0
US	2010	159334	93.3	100.0
WO	2010	23611	97.6	100.0

## CRediT Author Statement

**Gaétan de Rassenfosse:** Writing – Review & Editing, Supervision, Conceptualization. **Florian Seliger:** Software, Data Curation, Writing – Original Draft.

## Ethics Statement

Not applicable.

## Declaration of Competing Interest

The authors declare that they have no known competing financial interests or personal relationships which have, or could be perceived to have, influenced the work reported in this article.

## Footnotes

1 Sometimes, a patent document in office A can give rise to two or more patent documents in secondary office B (case of split applications). Conversely, two or more patent documents in office A can be merged into one application at office B.
